# Critical Care Experience With Clinical Cerebral Autoregulation Testing in Adults With Traumatic Brain Injury

**DOI:** 10.7759/cureus.43451

**Published:** 2023-08-14

**Authors:** Thitikan Kunapaisal, Monica S Vavilala, Anne Moore, Marie A Theard, Abhijit V Lele

**Affiliations:** 1 Anesthesiology and Pain Medicine, University of Washington, Seattle, USA; 2 Anesthesiology, Prince of Songkla University, Songkhla, THA; 3 Cerebrovascular Laboratory, Harborview Medical Center, Seattle, USA; 4 Anesthesia and Critical Care, University of Washington, Seattle, USA

**Keywords:** severe, moderate, tbi, traumatic brain injury, clinical practice, complications, safety, adults, cerebral autoregulation, transcranial doppler

## Abstract

Background: To describe the setting, feasibility, and safety of static cerebral autoregulation testing in critically injured adults with traumatic brain injury (TBI).

Methods: We reviewed static autoregulation testing using transcranial Doppler (TCD) ultrasound in patients > 18 years with TBI ICD codes between January 1, 2014, and December 31, 2021. Adverse events during testing were defined as systemic hypertension (systolic blood pressure (SBP>180 mmHg), bradycardia (HR<40 bpm), and high ICP (>30 mmHg). Impaired and absent cerebral autoregulation was defined as an autoregulatory index (ARI) <0.4 and ARI 0, respectively. We characterized prescribed changes in intracranial pressure (ICP) and cerebral perfusion pressure (CPP) targets by autoregulation testing results.

Results: A total of 135 patients, median age 31 (interquartile range (IQR) 24, 43) years, 71.9% male, admission Glasgow coma scale (GCS) score 3 (IQR 3, 5.5), and 70.9% with subdural hematoma from severe (GCS 3-8; 133 (98.5%)) and moderate (GCS 9-12; 2 (1.5%)) TBI, underwent 309 attempted testing. All patients were mechanically ventilated and had ICP monitoring; 246 (80%) had brain tissue oxygen monitoring, and 68 (22%) had an external ventricular drain. The median number of autoregulation tests was two (range 1-3) tests/patient, and the median admission to the first test time was two days (IQR 1, 3). Of 55 (17.8%) tests not completed, systemic hypertension (32, 10.4%), intracranial hypertension (10, 3.2%), and bradycardia (3, 0.9%) were transient. Fifty-three (51%) of the first (n=104) autoregulation tests showed impaired/absent cerebral autoregulation. Impaired/absent autoregulation results at the first test were associated with repeat cerebral autoregulation testing (RR 2.25, 95% CI [1.40-3.60], p=0.0007) than intact cerebral autoregulation results. Pre-testing cerebral hemodynamic targets were maintained (n=131; 86.8%) when cerebral autoregulation was impaired (n=151; RR 1.49, 95% CI [1.25-1.77], p<0.0001). However, 15 (9.9%) test results led to higher ICP targets (from 20 mmHg to 25 mmHg), 5 (3.3%) results led to an increase in CPP target (from 60 mmHg to 70 mmHg), and five out of 131 (3.8%) patients underwent decompressive craniectomy and placement of an external ventricular drain. Intact cerebral autoregulation results (n=43/103, 41.7%) were associated with a change in ICP targets from 20 mmHg to 25 mmHg (RR 3.15, 95% CI [1.97-5.03], p<0.0001).

Conclusions: Static cerebral autoregulation testing was feasible, safe, and useful in individualizing the care of patients with moderate-severe TBI receiving multimodal neuromonitoring. Testing results guided future testing, cerebral hemodynamic targets, and procedural decisions. Impaired cerebral autoregulation was very common.

## Introduction

Traumatic brain injury (TBI) is a global health concern. According to the Centers for Disease Control and Prevention (CDC), there were approximately 223,135 TBI-related hospitalizations in 2019 and 64,362 TBI-related deaths in 2020, which represents more than 611 TBI-related hospitalizations and 176 TBI-related deaths per day in the US [1].

Cerebral autoregulation is a hemostatic process that regulates and maintains cerebral perfusion pressure (CPP) and cerebral blood flow (CBF) across a range of blood pressures. Systemic hypotension leads to cerebral vasodilation to maintain CBF, which in turn elevates intracranial pressure (ICP) [2]. Conversely, systemic hypertension will cause cerebral vasoconstriction to maintain normal CBF and ICP. Consequently, if cerebral autoregulation is impaired, the cerebral circulation is "pressure passive." Prior studies demonstrate that impaired autoregulation may be associated with the worst clinical outcomes [3-5]. Factors that affect cerebral autoregulation are ICP (25 mmHg), lower arterial blood pressure (ABP<75 mmHg), or higher ABP (>125 mmHg) [6]. Cerebral autoregulation limits may affect optimal cerebral perfusion pressure (CPP), which in turn may be associated with favorable clinical outcomes [7,8]. Cerebral autoregulation testing results also have the potential to guide clinical care, but there is minimal information on how TCD autoregulation testing results are used clinically. There is also sparse literature on feasibility and safety and clinical settings under which TCD autoregulation testing is performed in adults with TBI [9,10].

Transcranial Doppler (TCD) ultrasound examinations are performed daily to assess the presence and severity of cerebral vasospasm in patients with spontaneous subarachnoid hemorrhage [11], but cerebral autoregulation testing is not routinely performed in patients with traumatic subarachnoid hemorrhage. Currently, TCD is not routinely used, cerebral autoregulation testing is not routinely considered part of advanced neuromonitoring, and there is no clinical protocol for cerebral autoregulation testing in patients with TBI. Given the availability of TCD technology and cerebral autoregulation testing expertise, we posited that clinical autoregulation testing may be clinically used and beneficial in patients with TBI and, hence, described 1) clinical setting, 2) feasibility, 3) safety, and 4) impact of testing results in adult patients with TBI admitted to our critical care unit.

## Materials and methods

Study setting 

This retrospective study was performed at Harborview Medical Center (HMC), a 413-bed, Level I adult and pediatric trauma center in Pacific Northwest. Inclusion criteria were adult patients 18 years admitted between January 1, 2014, and December 31, 2021, with moderate (admission Glasgow coma scale (GCS) score of 9-12) or severe (admission GCS 3-8) TBI, with or without polytrauma. Exclusion criteria were stroke patients and brain aneurysms. 

Static TCD cerebral autoregulation testing procedure 

The HMC cerebrovascular laboratory at HMC performs TCD monitoring and participates in cerebral autoregulation testing per protocol if the test is ordered by the treating teams. Cerebral autoregulation studies are performed by one certified cerebrovascular laboratory technologist. An electronic order set [12] details the desired type of cerebral autoregulation testing (static or dynamic), as well as baseline and testing boundaries and conditions. Given the large number of patients with TBI who have polytrauma, static cerebral autoregulation testing is preferred to thigh cuff methods [13].

Standard TCD monitoring protocols are used to monitor flow velocities, typically of the middle cerebral arteries (VMCAs). Before starting cerebral autoregulation testing, the TCD technician notes contemporaneous mean arterial pressure (MAP), heart rate (HR), ICP, cerebral perfusion pressure (CPP), partial pressure of carbon dioxide (PaCO2), GCS at the time of testing, serum hematocrit, and serum sodium. Baseline MCA mean flow velocities, ICP, and CPP are recorded. After obtaining baseline values, patients are administered a vasoactive agent to raise the MAP to achieve the CPP target, which is typically 20% higher CPP than the baseline. Careful attention is paid to the ICP as well as HR during changes in CPP. Once target CPP is achieved, bilateral MCA mean flow velocities are measured.

Cerebral autoregulation testing frequency and the decisions taken after the results of TCD autoregulation studies are not protocolized and are left to the discretion of the multidisciplinary clinical management team of neurosurgeons and an intensivist.

Data collection 

We abstracted clinical data, such as age, sex, race, mechanism of injury, abnormalities on admission computerized tomography of the head (CT), polytrauma by organ system affected, admission GCS, duration of intensive care unit and hospital stay, and discharge disposition. We evaluated the total number of cerebral autoregulation tests performed and vital signs, such as MAP, HR, ICP, CPP, and PaCO2 at the time of cerebral autoregulation testing, as well as contemporaneous data from the brain tissue oxygenation monitor.

We examined the management of elevated ICP such as sedation, hyperosmolar treatment such as hypertonic saline/mannitol, and vasoactive medication during or after TCD autoregulation testing. We reviewed bedside nurses/intensivist/neurosurgical service progress notes for documentation of cerebral hemodynamic goals before and after TCD autoregulation testing.

Outcomes 

Primary outcomes were 1) clinical setting of cerebral autoregulation testing use; 2) feasibility of testing, defined as a percentage of completed cerebral autoregulation tests; 3) adverse events, defined as absence of unwanted changes in hemodynamics during testing such as elevated ICP (>30 mmHg), systolic hypertension (SBP>180 mmHg), and bradycardia (HR<40 bpm); and 4) changes in cerebral hemodynamic goals after cerebral autoregulation testing.

ARI calculations were made by certified institutional TCD technologists. The autoregulation index (ARI) is calculated using the following formula: ARI=%ΔeCVR/%ΔMAPe, where eCVR is the estimated cerebrovascular resistance calculated as the ratio of MAP to VMCA as appropriate. ARI values < 0.4 indicate impaired autoregulation, and values 1 indicate intact cerebral autoregulation [14].

Data analysis 

Categorical variables, such as sex, race, and mechanism of injury, are described as numbers and percentages. Continuous variables, such as age, GCS, and length of hospital stay (LOS), are reported as median and interquartile ranges (Q3-Q1) or mean and standard deviation (SD). Categorical variables were compared by using the chi-square test, relative risk, and 95% CI. A p-value < 0.05 indicated statistical significance. STATA 15 [15] and RStudio 1.554 [16] were used for statistical analysis.

## Results

During the study period, 5,665 patients with TBI GCS 3-12 were admitted; of these, 428 had multimodal neuromonitoring (ICP and cerebral tissue oxygen partial pressures (PBO2)). No patient without advanced neuromonitoring underwent TCD autoregulation testing compared to 135/428 (31%) of those with advanced neuromonitoring.

Patient characteristics

As Table 1 shows, 135 patients underwent cerebral autoregulation testing during the study period.

**Table 1:** Characteristics of the patients with traumatic brain injury who underwent cerebral autoregulation testing with transcranial Doppler ultrasonography Data are presented as numbers (%), unless indicated otherwise. IQR=interquartile range

Patient characteristics	n=135
Age (years), median (interquartile range, IQR)	31 (24,43)
Male, n (%)	97 (71.9%)
Caucasian race/ethnicity, n (%)	102 (75.6%)
Mechanism of injury, n (%)	
Motor vehicle collision	80 (59.3%)
Fall	33 (24.4%)
Crush/blunt	13 (9.6%)
Firearm	9 (6.7%)
Polytrauma, n (%)	104 (77.03%)
Thorax	71 (68.3%)
Extremities	54 (51.9%)
Abdomen	27 (26.0%)
Admission Glasgow coma scale score, median (IQR)	3 (3,5.5)
Abnormalities on computerized tomography of the brain, n (%)	
Subdural hematoma	95 (70.4%)
Subarachnoid hemorrhage	93 (68.9%)
Cerebral contusion	69 (51.1%)
Skull fracture	57 (42.2%)
Intraparenchymal hemorrhage	40 (29.6%)
Epidural hematoma	22 (16.3%)
Pneumocephalus	15 (11.1%)
Intensive unit length of stay, median (IQR)	30 (17,48)
Hospital length of stay, median (IQR)	30 (17,48)
Tracheostomy, n (%)	33 (24.4%)
Gastrostomy feeding tube, n (%)	47 (34.8%)
Discharge Glasgow coma scale score, median (IQR)	14 (5,15)
Discharge disposition, n (%)	
Transfer to a rehabilitation facility	52 (38.5%)
Expired	37 (27.4%)
Transfer to a skilled nursing facility	24 (17.8
Home	17 (12.6%)
Transfer to the acute care facility	4 (3%)
Against medical advice	1 (0.7%)

Clinical setting 

During the study period, 309 static autoregulation tests were attempted in 135 patients (median number of tests per patient 2; a range of 1-3 tests per patient (Table 2).

**Table 2:** Clinical setting during transcranial Doppler ultrasonography-based cerebral autoregulation Data are presented as numbers (%), unless indicated otherwise. TCD=transcranial Doppler

Clinical setting	Total tests (n=309)
Time from admission to first autoregulation test in days, median (IQR)	2 (1,3)
Glasgow coma scale score at the time of testing, median (IQR) (n=306)	4 (3,7)
Cerebral hemodynamic profile	
Heart rate (bpm), median (IQR) (n=308)	79 (67,92)
Mean arterial blood pressure (mmHg), median (IQR) (n=303)	84 (79,90)
Cerebral perfusion pressure (mmHg), median (IQR) (n=308)	73 (65,83)
Partial pressure carbon dioxide (torr), median (IQR) (n=307)	37 (35,40)
Intracranial pressure (mmHg), median (IQR)	16 (11,20)
Mechanically ventilated	309 (100%)
Monitoring	
Invasive arterial blood pressure	309 (100%)
Intracranial pressure	309 (100%)
External ventricular drain	68 (22.0%)
Electroencephalography	289 (93.5%)
Brain tissue oxygenation	246 (80.0%)
Pharmacotherapy	
Vasoactive medication	270 (87.4%)
Sedation and analgesia	258 (83.5%)
Hyperosmolar therapy (hypertonic saline/mannitol)	192 (62.1%)
Neurosurgical intervention performed prior to the TCD autoregulation test	
Decompressive craniectomy	65 (21.0%)
Craniotomy	14 (4.5%)

Feasibility of use and safety 

**Figure 1:**
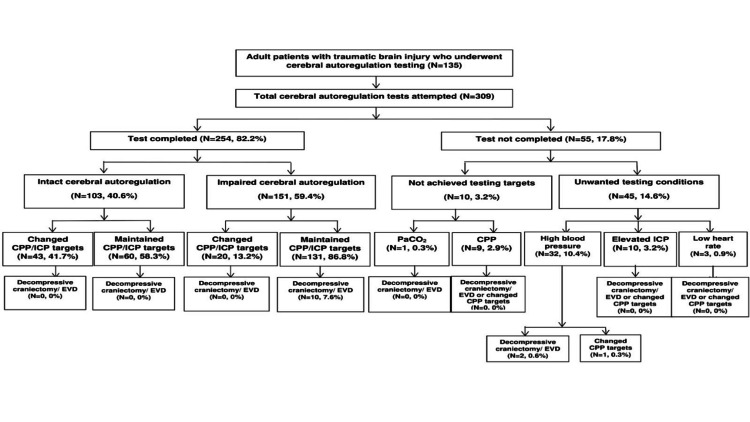
Performance of cerebral autoregulation assessments in adults with traumatic brain injury ICP=intracranial pressure; CPP=cerebral perfusion pressure; EVD=external ventricular drain

Figure 1 shows that cerebral autoregulation tests were completed in 254 (82.2%). Fifty-five attempts (17.8% tests) were aborted either due to patient factors bradycardia (HR<40 bpm) (n=3/309, 0.9%), high blood pressure (SBP>180 mmHg) (n=32/309, 10.4%), and elevated ICP (>30 mmHg) (n=10/309, 3.2%); all of which were transient and did not require medical intervention, as well as technical factors (unable to reach the target CPP (n=9/309, 2.9%) and PaCO2 (n=1/309, 0.3%) targets.

Clinical actions after the first cerebral autoregulation test result 

Patients with the First Intact Cerebral Autoregulation 

Patients with first intact cerebral autoregulation (n=43, 41.7%) were more likely (RR 3.15, 95% CI [1.97-5.03], p<0.0001) to experience a change in cerebrovascular target than patients with impaired cerebral autoregulation on testing from the original target of 20 mmHg to a new target of 25 mmHg. 

*Patients with the First Impaired/Absent Cerebral Autoregulation* 

Fifty-three (51%) of 104 completed first cerebral autoregulation tests demonstrated impaired/absent cerebral autoregulation. Impaired/absent autoregulation results at the first test led to repeat cerebral autoregulation testing (RR 2.25, 95% CI [1.40-3.60], p=0.0007) compared to intact cerebral autoregulation results. Pre-testing cerebral hemodynamic targets were maintained (n=131; 86.8%) when cerebral autoregulation (n=151) was impaired (RR 1.49, 95% CI [1.25-1.77], p<0.0001), but some patients (n=5/131, 3.8%) underwent decompressive craniectomy and placement of an external ventricular drain (n=5/131, 3.8%). Some test results (n=15/151, 9.9%) led to higher ICP targets (from 20 mmHg to 25 mmHg), and five test results (3.3%) resulted in a change in the CPP target from 60 mmHg to 70 mmHg.

Clinical outcome

At the time of discharge, the median Glasgow outcome scale-extended (GOSE) was 3 [range 2-3] and 3 [range 1-3] for intact and impaired CA, respectively.

## Discussion

This study examined the clinical setting, safety, feasibility, and clinical use of static cerebral autoregulation assessment using TCD technology in adults with moderate-severe TBI, who underwent multimodal neuromonitoring in the intensive care unit. The main findings are as follows: 1) cerebral autoregulation tests often demonstrated impaired cerebral autoregulation; 2) clinicians ordered repeat cerebral autoregulation tests when the first test results showed impaired cerebral autoregulation; 3) cerebral autoregulation testing was safely performed; and 4) the results of cerebral autoregulation testing, whether intact or impaired, informed cerebral hemodynamic targets and procedural decisions. Together, these findings suggest that cerebral autoregulation testing may benefit the care and outcomes of patients with moderate-severe TBI.

Our study finds that cerebral autoregulation testing can be safely performed in patients with moderate-severe TBI. The fact that 17.8% of tests were not completed due to systemic hypertension as pharmacologic agents were used to increase MAP/CPP, and high ICP during testing reflects impaired cerebral autoregulation. Three patients had bradycardia; together this means that the cerebral autoregulation test can also serve as a provocative cerebral hemodynamic stability test to guide medical and procedural decisions, such as time to surgery. The 80% testing completion rate may be lower than desired, but testing was performed in the most injured patients, suggesting a selection bias even among patients who fall within the category of moderate-severe TBI.

Testing failure rates fell into two categories (patient and technical). The technical failure rates of failing to achieve desired hemodynamic targets are often beyond testing condition control and do not cause patient harm. Patient factors, such as systemic hypertension, bradycardia, and high ICP, were transient and required no medical intervention. However, we think that testing environments should include plans for treating sustained or severe hypertension, high ICP, and bradycardia and that rates of adverse events are consistent with our prior experience [12]. Static cerebral autoregulation testing is a provocative challenge test that appears to add additional value to multimodal neuromonitoring, and this deserves further study.

All patients who underwent cerebral autoregulation testing had advanced neuromonitoring, which does not include a functional assessment of cerebrovascular stability. Our experience suggests that static cerebral autoregulation testing reflects cerebral hemodynamic instability rather than being a problem with testing. In this study, impaired cerebral autoregulation on initial examination was associated with repeat testing, suggesting our clinicians used results to guide testing and care given that normalization of autoregulation status in severe TBI may be prolonged [17]. While we cannot definitively ascertain this from our design, since aborted testing, our data from patients who underwent repeat testing suggests that repeat testing may be attempted to understand cerebral hemodynamic recovery. The utility of repeat testing in the first week after TBI underscores the recognition of derangements in cerebrovascular physiology and obtaining valuable data points that helped clinicians in individualizing the care of patients with moderate-severe TBI.

We observed a temporal association between cerebral autoregulation results and decompressive craniectomy/external ventricular drain placement. At our institution, decompressive craniectomy (primary or secondary) [18] is typically performed for patients with intracranial hypertension, and external ventricular drains are used for intermittent cerebrospinal fluid drainage (also known as "burping" of cerebrospinal fluid). Kolias et al. [19] demonstrated in a case series that decompressive craniectomy after TBI led to a reduction in ICP and improved CBF. Similar results were reported by Wang et al. [20], who demonstrated improved pressure-reactivity index in patients who had undergone a decompressive craniectomy. We hypothesized that external ventricular drain by decompressing the cerebrospinal compartment may also improve cerebral autoregulation. Our data suggest that clinicians used cerebral autoregulation test results to guide procedural care. The finding that cerebral autoregulation testing results that guide cerebral hemodynamic targets is important. Our data show that clinicians tolerated higher CPP and ICP targets when cerebral autoregulation was intact. Conversely, when autoregulation studies demonstrated impaired cerebral autoregulation, clinicians more often than not continue with the same cerebral hemodynamic targets. As shown in Figure 1, clinician behavior was not universal for these patients as not every patient with intact or impaired cerebral autoregulation had modification/continuation of cerebral hemodynamic targets. This implies clinicians used testing results to individualize the approach to patient management.

This study has some limitations and strengths. First, we examined our clinical practice over eight years, when documentation of results and actions taken because of test results were not standardized. While the study team reviewed clinical records, this may have introduced errors in estimates and types of decisions attributed to testing results. Second, we were unable to determine specific interventions that led to a change in PBO2 in the non-advanced neuromonitoring group because there was no specific timestamp to evaluate, and specific interventions were not consistently documented. Lastly, our sample size is small and from a single center. The strengths of this study are standardization of cerebral autoregulation testing, testing performed by certified experts in a center with experience in cerebral autoregulation testing, and detailed analyses of testing conditions and outcomes. Despite these limitations, this first account of how clinicians use cerebral autoregulation testing results in TBI provides new information on how cerebral autoregulation testing can serve as an important provocative test of cerebral hemodynamic stability and guide clinical care.

## Conclusions

Cerebral autoregulation testing was feasible, safe, and clinically useful in the care of patients with moderate-severe TBI receiving multimodal neuromonitoring in an experienced center. Over half of the cerebral autoregulation tests demonstrated impaired cerebral autoregulation. Cerebral autoregulation results led to testing decisions, informed clinician understanding of cerebral hemodynamic stability, and guided cerebral hemodynamic targets and procedural decisions in many patients.
